# Evaluation of Serial Thawing-Refreezing on Human Spermatozoa Resistance Using Cryovials and Straws

**Published:** 2012-12-17

**Authors:** Fatemeh Ghasemian, Roya Faraji, Mohaddese Mohammadi Sardoo, Mohammad Hadi Bahadori

**Affiliations:** 1 Biology Faculty, Kharazmi University, Tehran, Iran; 2 Infertility Therapy Center, Al-Zahra Educational and Remedial Center, Guilan University of Medical Sciences, Rasht, Iran; 3 Cellular and Molecular Research Center, Faculty of Medicine, Guilan University of Medical Sciences, Rasht, Iran

**Keywords:** Semen, Oligozoospermia, Freezing- Thawing

## Abstract

**Background:**

We designed this study to detect the cryoinjury rate on human sperm after serial freezing and thawing, taking into consideration the effects of using cryovials and straws.

**Materials and Methods:**

In this experimental study, semen specimens obtained from 15 subjects were divided into normozoospermic and oligozoospermic groups. Each of the normozoospermic and oligozoo spermic semen specimens were additionally divided into two groups: i. washed and ii. unwashed. Specimens were repeatedly freeze-thawed by using cryovials and straws with the fast liquid nitrogen vapor method, until no motile sperm remained. Sperm motility, recovery, and morphology rate were then determined after thawing, and compared between the groups while taking into consideration the effects of using cryovials and straws.

**Results:**

Motile spermatozoa were observed in all normozoospermic samples up to thaw 6 with both cryovials and straws while in oligozoospermic specimens up to thaw 4 (straw) and thaw 3 (cryovial) in the freeze-thawing cycle. Normozoospermic sample analysis showed no significant difference in morphology rate. There was a significant increase in motility and recovery percentages for washed samples, which was observed with straws in compared to the unwashed groups. Oligozoospermic sample analysis indicated a significant increase in motility, recovery (p<0.01), and morphology (p<0.001) rates in washed specimens compared to unwashed specimens using straws. The importance of washing sperm was obvious for oligozoospermic specimens.

**Conclusion:**

Normozoospermic sperm resisted freezing longer than oligozoospermic sperm. Use of straws and cryovials made significant differences in motility, recovery, and morphology of sperm in each thaw. This difference was slightly higher for oligozoospermic specimens. Results indicated that the percentage of motility was higher for washed normozoospermic specimens in each thaw when straws were used, whereas the percentage of motility, recovery, and morphology were promoted after frozen oligozoospermic specimens were washed using straws.

## Introduction

Sperm cryopreservation is important for men who have faced certain problems, such as difficulty producing semen during donor and assisted reproductive technology (ART) programs, cancer and chemotherapy, radiotherapy, vasectomy (1 , 2 ) and surgical treatments (3 ). The success rate of pregnancy per attempt is below 50% (4 ) and thus many couples need multiple ART attempts in order to achieve a successful pregnancy. ART procedures, particularly intracytoplasmic sperm injection (ICSI), require some samples of thawed/fresh sperm. In these procedures, the remaining sperm are discarded following each cycle (1 , 5 ). Freeze-thawed specimens that are refrozen would provide additional opportunities for conception without compromising their ability to be used for infertility treatments. Thus cryopreservation is often recommended to preserve future fertility (3 ).

The freeze-thaw process significantly decreases sperm survival and motility (3 ), acrosome integrity (6 ), the ability of sperm to penetrate into the cervical mucus (7 ), and induces sperm apoptosis (8 ). The improvement of human spermatozoa cryopreservation leads to better thawed gametes (5 ). It has been reported that ICSI using both frozenthawed and fresh motile spermatozoa obtain similar fertilization and pregnancy rates (4 ).

Sperm quality and quantity are often influenced by severe oligoasthenoteratozoospermia and testicular cancer. Therefore, conservation of even a few sperm for ICSI after serial freeze-thaw cycles is important (3 ). Refrozen sperm is beneficial for those who have difficulty producing semen, thus it is important for men to produce further samples during subsequent ART cycles (1 ). Verza et al. have reported the recovery of motile spermatozoa after five refreeze-thawing cycles (for normozoospermic) and two refreezethawing cycles (for oligozoospermic) specimens that had been frozen by using a vial cryovial (5 ). The percentage of motility for rapid freeze-thawed normozoospermic samples that used a straw has been reported as 47.4% (unwashed) and 67.1% (washed); morphology was reported as 95.4% (unwashed) and 84.9% (washed). These percentages in oligozoospermic specimens were 22.3% (unwashed) and 33.3% (washed) for motility and 70.9% (unwashed) and 87.6% (washed) for morphology (1 ). In frozen normozoospermic samples that used a 1.0 ml cryovial, motility was 50.6% and the recovery rate was 78.0%. In oligozoospermic samples motility was 11.9% and the recovery rate was 20.7% (5 ).

The purpose of this study was to evaluate the resistance rate of human normozoospermic and oligozoospermic specimens by the fast liquid nitrogen vapor method until no motile sperm were observed after serial refreeze-thaw cycles. The effects of using cryovials and straws, and the presence of seminal plasma were also considered.

## Materials and Methods

### Semen collection and sperm assessment

This experimental study, was approved by the Ethical and Scientific Committee of Guilan University of Medical Sciences (GUMS). Semen samples were obtained from couples who referred to the Al-Zahra Infertility Therapy Center and informed consent was obtained from these patients. The remaining specimens were analyzed and used after obtaining patients' informed consents for scientific use. Semen samples were collected by masturbation after 3-4 days of ejaculatory abstinence into sterile containers and were allowed to liquefy in an incubator at 37°C for 30 minutes. An aliquot of each liquefied specimen was analyzed according to World Health Organization guidelines (9 ).

The search strategy retrieved 30 potential samples and subsequently included 15 samples with the following criteria: patient age (26-32 years), sperm concentration (>0.5×10^6^/mL), sperm morphology (>20%), motility (>40%), progression (>20%), and leukocyte (0 to 5×10^6^/mL) rates. According to the pre-freeze sperm count, at the time of freezing, there were eight specimens classified as normozoospermic (≥20×10^6^/mL) and seven oligozoospermic (<20×10^6^/mL) specimens. Normozoospermic and oligozoo-spermic specimens were further subdivided into two groups of semen: i. washed and ii. unwashed. Specimens from each group were separately vitrified by using cryovials or straws.

### Cryopreservation and thawing procedures

Semen samples were allowed to liquefy in an incubator at 37°C for 30 minutes after collection. Washed spermatozoa were prepared with a density- gradient separation medium (two-step gradient of 90% and 45%; SpermGrad, Vitrolife, Sweden). To delete the remaining dead sperm, the swim up method was performed. The final washed specimens were resuspended in sperm washing medium (G-IVF™ PLUS, Vitrolife, Sweden). Both washed and unwashed specimens were cryopreserved using freezing medium (Quinn’s Advantage Sperm Freeze, USA) and vitrified by the fast liquid nitrogen vapor method, according to the company's protocol. One volume of freezing medium was added, drop by drop over a 30-second period to one volume of liquefied semen or washed spermatozoa suspension. This process was repeated and the final ratio of freezing media to semen/spermatozoa suspension was 1:1 (v/v). The mixture was equilibrated for 3 minutes, then loaded into 1.0 ml cryovials and 0.5 ml straws (MVE, France). Cryovials and straws were transferred quickly to liquid nitrogen vapor at the top of a liquid nitrogen storage tank and left for 30 to 45 minutes. Both cryovials and straws were then plunged into liquid nitrogen for storage at -196°C. For thawing, all cryovials and straws from each group were removed from the liquid nitrogen storage tank and thawed at room temperature for 10 minutes. Cryovials and straws were then transferred to a 37°C incubator for 10 minutes (10 , 11 ).

### Assessment of sperm parameters

Routine semen analysis was performed by light microscopy according to World Health Organization criteria (9 ). Motility, morphology analysis and the percentage of thawed sperm from each individual patient were carried out after each thaw as follows (1 ):

$$\frac{\mathrm{Motility (\%) of the thawed sample}}{\mathrm{Motility (\%) of the pre-frozen sample}}\times 100$$

$$\frac{\mathrm{Morphology (\%) of the thawed sample}}{\mathrm{Morphology (\%) of the pre-frozen sample}}\times 100$$

Morphology analysis was performed based on the following criteria: sperm size, the presence of a cytoplasmic neck, and acrosome. In this manner, the sperm cells were suspended in a microdrop. The sterile glass bottom dish that contained the microdrop was then placed on a microscope stage to be examined at high magnification by an inverted microscope (Olympus IX70, Tokyo, Japan). The total calculated magnification was ×6600 (12 ).

### Repeated freeze-thawing procedure

After removing an aliquot for analysis, we used the same freezing method as described above to perform repeated freezing. In this way, the cryoprotectant content of the original freezing cycle did not change. The specimens were then stored in liquid nitrogen for at least 48 hours and thawed using the method previously described. The thaw-refreeze cycles were repeated until no motile sperm were obtained.

### Statistical analysis

Data are expressed as mean ± SD. Pre- and postfreeze sperm parameters, percentage of motility, recovery of motile sperm, and spermatozoa morphology for normozoospermic and oligozoospermic groups after each thaw were calculated. Statistical analysis was performed using the SPSS 15.0 statistical package (SPSS). All outcomes were assessed using Chi-squared test and t test and with a significance level of p<0.05.

## Results

The mean ± SD age of normozoospermic individuals was 31.0 ± 1.1 years and for oligozoospermic individuals it was 28.7 ± 2.3 years. Pre-freeze sperm parameters are shown in Table 1. The mean percentage of the pre-freezing sperm concentration was 89.3 ± 33.6 × 10^6^, for motility it was 69.1 ± 12.8%, and morphology was 57.4 ± 16.1% for all unwashed eight normozoospermic samples. Of the seven oligozoospermic samples the mean pre-freezing sperm concentration was 10.4 ± 3.2 × 10^6^, motility was 53.6 ± 10.4%, and morphology was 41.7 ± 14.5%.

For analysis, we compared the motility, recovery (sperm concentration) and morphology rates of washed normozoospermic samples between frezed-thawed groups using straws and cryovials. The same analysis was done for unwashed normozoospermic groups. The best result in each comparison of the washed and unwashed samples was then compared. The mean ± SD for motility, recovery, and morphology percentages of washed and unwashed samples for the eight normozoospermic individuals are summarized for each thaw using straw and cryovial in table 2.

There were significant differences in motility, recovery, and morphology percentages between vitrified groups by straw and cryovial for each thaw in the washed group. Similar results were obtained for the unwashed groups. This difference increased for thaws 4 to 6 in the unwashed groups. Comparison of straw results between washed and unwashed groups indicated no significant differences in morphology rate. However a significant increase in motility percentage for washed samples was observed using the straw. Washed sperm samples had slightly higher recovery rates than unwashed samples in thaws 3 to 6, but was not statistically significant. Motile spermatozoa were observed in all normozoospermic samples up to thaw 6 of the refreeze-thawing cycle.

**Table 1:** Specimen characteristics before freezing in washed/ unwashed normozoospermic and oligozoospermic groups


	Normozoospermic (n=8)		Oligozoospermic (n=7)	
	Unwashed	Washed	Unwashed	Washed

**Sperm count (×10^6^/ml)**	89.3 ± 2.82	93.4 ± 4.8	10.4 ± 0.7	12.9 ± 1.41
**Motility (%) **	69.1 ± 3.25	71.4 ± 1.41	53.6 ± 0.8	54.6 ± 2.26
**Progression motility (%)**	52.8 ± 3.11	55.1 ± 0.14	34.8 ± 2.26	36.7 ± 1.41
**Leukocytes (×10^6^/ml)**	1.0 ± 0.07	NA*	0.8 ± 0.14	NA*
**Morphology (%)**	57.4 ± 2.26	58.5 ± 0.7	41.7 ± 3.11	43.5 ± 0.7


*NA; Not available. Values are mean ± SD.

Analysis of the motility, recovery, and morphology percentage for oligozoospermic specimens was similar to normozoospermic specimens. The mean ± SD for motility, recovery, and morphology percentages of washed and unwashed samples for the seven oligozoospermic individuals are summarized for each thaw using straws and cryovials in table 3. There was a significant increase in motility, recovery, and morphology rate in refrozen specimens using the straw in each group compared to the cryovial group (p<0.05). The results indicated a significant increase in motility, recovery, and morphology rates in washed specimens compared to unwashed specimens using straws (p<0.05). These differences were slightly higher in the oligozoospermic samples.

**Table 2:** Motility, recovery, and morphology percentage between washed and unwashed sperm specimens in the normozoospermic group using straw and cryovial after repeated refreezing-thawing


	Normozoospermic (n=8)			
	Washed		Unwashed	
	Straw	Cryovial	Straw	Cryovial

**1^st^ thaw**				
**Motility (%)**	62.3 ± 2.82	57.2 ± 3.11	55.8 ± 2.75^A^	46.3 ± 0.07
**Recovery (%)**	83 ± 2.82	78 ± 2.82	80.5 ± 0.7	72.9 ± 2.82
**Morphology (%)**	89.4 ± 3.94^C^	77.5 ± 2.4	92.1 ± 2.97	85.5 ± 0.7
**2^nd^ thaw**				
**Motility (%)**	41 ± 2.12C	29.9 ± 2.82	33.1 ± 4.38	25.8 ± 1.13
**Recovery (%)**	67.3 ± 2.82^B^	51 ± 1.41	64.9 ± 2.82	56.8 ± 2.75
**Morphology (%)**	67.9 ± 3.04	58.2 ± 3.11	70.2 ± 0.07	62.7 ± 2.82
**3^rd^ thaw**				
**Motility (%)**	24.4 ± 0.84^C, b^	16.4 ± 0.84	18.1 ± 1.27	13.7 ± 1.34
**Recovery (%)**	50.4 ± 5.86^C, b^	40.9 ± 0.42	43.2 ± 1.13^A^	35.5 ± 2.12
**Morphology (%)**	51.2 ± 1.13^C^	43.4 ± 0.84	53.2 ± 1.13^A^	46.7 ± 1.41
**4^th^ thaw**				
**Motility (%)**	15.3 ± 1.83^A, a^	6.9 ± 1.41	8.1 ± 0.14	5.1 ± 1.55
**Recovery (%)**	46.7 ± 1.84^B, a^	36.7 ± 1.41	40.9 ± 1.27^C^	30.2 ± 0.07
**Morphology (%)**	43 ± 1.41^B^	34.6 ± 1.34	44.1 ± 1.41^B^	34.9 ± 1.34
**5^th^ thaw**				
**Motility (%)**	11.9 ± 0.35^B, a^	3.8 ± 1.41	5.5 ± 1.41	1.8 ± 0.28
**Recovery (%)**	29.6 ± 1.13^B^	20.5 ± 1.41	24.7 ± 1.41^A^	17.7 ± 1.41
**Morphology (%)**	35.8 ± 1.2^C^	22.7 ± 1.41	37 ± 1.41^B^	25.9 ± 1.41
**6^th^ thaw**				
**Motility (%)**	3.9 ± 0.14^A^	0.4 ± 0.14	1.7 ± 0.0	0.1 ± 0.0
**Recovery (%)**	19.7 ± 0.49^C, a^	12 ± 1.41	15.3 ± 1.83^B^	6.4 ± 1.98
**Morphology (%)**	29 ± 1.41^C^	16.3 ± 0.42	31.7 ± 1.41^B^	21.1 ± 1.55


Values are mean ± SD. Comparison between thawed groups by straw and cryovial in both washed and unwashed specimens. A; p<0.05, B; p<0.01, and C; p<0.001. Comparison was also done between washed and unwashed specimens using straw: a; p<0.05, b; p<0.01, and c; p<0.001.

**Table 3:** Motility, recovery, and morphology percentage between washed and unwashed sperm specimens in the oligozoospermic group using straw and cryovial after repeated refreezing-thawin


	Normozoospermic (n=8)			
	Washed		Unwashed	
	Straw	Cryovial	Straw	Cryovial

**1^st^ thaw**				
**Motility (%)**	17.9 ± 1.41	13.8 ± 1.83	15.3 ± 0.42^B^	11 ± 0.07
**Recovery (%)**	48.2 ± 1.27^B, b^	38.7 ± 1.9	39.2 ± 1.41^A^	35.4 ± 0.84
**Morphology (%)**	67.9 ± 1.48^C^	48.9 ± 1.26	64.2 ± 0.14^C^	50.8 ± 0.56
**2^nd^ thaw**				
**Motility (%)**	13.8 ± 0.84^A^	8.1 ± 0.14	10 ± 0.07	6.3 ± 0.42
**Recovery (%)**	30 ± 1.41^A,a^	25.9 ± 1.27	23.5 ± 2.12	18.3 ± 1.83
**Morphology (%)**	48.3 ± 1.41^C, b ^	33.9 ± 0.07	36.2 ± 1.69^A^	30.2 ± 0.07
**3^rd^ thaw**				
**Motility (%)**	8.6 ± 0.84^A^	2.3 ± 0.42	5.3 ± 1.83	0.4 ± 0.14
**Recovery (%)**	18.7 ± 0.84^A, b^	12.4 ± 1.98	11.9 ± 0.14	7.3 ± 0.1
**Morphology (%)**	36.3 ± 0.7^C, c^	25.2 ± 1.69	22.3 ± 2.54	19.7 ± 0.14
**4^th^ thaw**				
**Motility (%)**	1.4 ± 0.56^a^	NA	0.6 ± 0.03	NA
**Recovery (%)**	10.5 ± 0.0^b^	NA	2.9 ± 0.14	NA
**Morphology (%)**	19.7 ± 0.56^b^	NA	11.4 ± 0.14	NA
**5^th^ thaw**				
**Motility (%)**	NA	NA	NA	NA
**Recovery (%)**	NA	NA	NA	NA
**Morphology (%)**	NA	NA	NA	NA
**6^th^ thaw**				
**Motility (%)**	NA	NA	NA	NA
**Recovery (%)**	NA	NA	NA	NA
**Morphology (%)**	NA	NA	NA	NA

Values are mean ± SD. Comparison between thawed groups by straw and cryovial in each washed and unwashed specimens. NA; Not available, A; p<0.05, B; p<0.01, and C; p<0.001. Comparison between washed and unwashed specimens using straw: a; p<0.05, b; p<0.01 and c; p<0.001.

There were motile spermatozoa in all oligozoospermic samples up to the third refreeze-thawing cycle using cryovials, while motile spermatozoa were observed up to the fourth thaw in the group vitrified by straws. These results indicated that the resistance of spermatozoa was higher when straws were used. The importance of washing sperm was evident for oligozoospermic specimens.

## Discussion

Studies focusing on the resistance of human spermatozoa to cryoinjury after repeated thawrefreezing cycles are scarce. Polcz et al. (13 ) have studied normozoospermic men and demonstrated the ability of human spermatozoa to resist cryoinjury in successive thaw-refreeze cycles. The spermatozoa were able to withstand five thaw-refreeze cycles and still maintain motility and vitality, although a marked reduction in motility occurred. In the thaw-refreeze cycles, they observed the following percentages of motile sperm: 3.5% (3rd cycle), 1.5% (4th cycle) and 1.8% (5th cycle). Despite a linear decrease in motility per cycle, spermatozoa resistance has been reported in up to seven cycles of the thaw-refreeze process. A comparison between slow and fast freezing techniques showed a 2.75 cycle ratio of preserved motility in fast freezing (3 ). Verza et al. (5 ) assayed the quality of sperm specimens using the fast liquid nitrogen vapor method for the initial freezing cycle. Bandularatne and Bongso (1 ) evaluated the extent of sperm cryoinjury in up to three repeated freezings in normozoospermic and oligozoospermic men using both slow and fast freezing techniques. They observed a significant reduction in the recovery of motile and viable sperm after each thaw, independent of the freezing methods.

Evaluation of resistance to cryoinjury in normal and weak sperm samples was carried out using different methods by Bandularatne and Bongso (1 ) and Verza et al. (5 ). In this way, repeated freeze-thawing was performed until no post-thaw motile sperm were visualized. Resistance spermatozoa were also compared between vitrified groups for washed and unwashed specimens using 0.5 ml straw and 1.0 ml cryovial methods. However, the methodology employed by Verza et al. (5 ) has differed from this study in that they only compared the rate of resistance of normozoospermic and oligozoospermic sperm; they did not assay the effect of the presence or absence of seminal plasma in washed and unwashed samples, and did not compare the effects of using cryovials and straws.

We observed that normozoospermic sperm resisted freezing longer than oligozoospermic sperm. Using either straws or cryovials made significant differences in sperm motility, recovery, and morphology. This difference was slightly higher for oligozoospermic sperm. Oligozoospermic resistance increased when freezing was done by straws up to thaw 4. Our results also indicated that the percentage of motility was higher for washed normozoospermic samples, while the percentage of motility, recovery, and morphology increased after washed oligozoospermic freezing using straws. We observed that the recovery of motile spermatozoa was significantly impaired after each refreeze-thawing cycle using cryovial for unwashed specimens.

Motile sperm were still present following five refreezing cycles for the normozoospermic group and three refreezing cycles for the oligozoospermic groups, both using straws. By using cryovials, motile sperm were observed at five refreezing cycles for normozoospermic groups and two refreezing cycles for oligozoospermic groups. Therefore, the normozoospermic specimens have shown more resistance than the oligozoospermic ones. Given that sperm cryo-survival is partially dependent on the semen quality before freezing (14 , 15 ), we have observed that spermatozoa with low quality is more sensitive to cryoinjury than normal spermatozoa. The quality and concentration of sperm can be influenced by stress during ART cycles (16 , 17 ).

The success rate of human sperm cryopreservation was influenced by important agents such as cryoprotectant, the freezing technique, and the initial quality of the specimen (5 ). For example, it was reported that the vitrification of mouse germinal vesicle oocytes using cryotop was more effective than straws (18 ). This observation has shown that a successful vitrification rate can be influenced by different agents. There is not any difference between slow freezing and fast freezing in thaw survival both in normal and poor quality sperm (17 ).

In this study, we used a rapid vapor freezing method because it was less expensive, time-consuming, and labor intensive, and it has proven equally effective in the recovery of post-thaw motile sperm (17 , 19 ). In our study, cryopreservation of specimens was performed with and without seminal plasma, and we did not change the cryoprotectant during the experiment. We have also examined the effects of removing the seminal plasma before freezing on sperm motility and morphology.

Thomson et al. (20 ) reported that the presence of fresh cryoprotectant in each freeze increased the level of DNA fragmentation (more than double), whereas the original cryoprotectant caused only a slight increase. In another study by the same authors, the percentage of sperm DNA fragmentation increased post-cryopreservation both with and without the addition of cryoprotectant (21 ). Their results indicated that the generation of DNA damage during cryopreservation was not influenced by cryoprotectant. Therefore, the washing steps could cause mechanical damage to cellular structures and possibly to the DNA molecule (4 , 22 ).

## Conclusion

Human spermatozoa resistance rate can differ when the serial refreeze-thawing cycle is performed with the cryovial and straw using the fast vapor freezing method. The percentage of recovery, morphology, and sperm motility have increased in the groups vitrified with straw. Vitrified oligozoospermic samples with straws could be more resistant (one cycle) than vitrified samples using cryovials. The washed specimens also showed better survival rate than unwashed ones. The importance of washing sperm was obvious for the oligozoospermic specimens. Many factors and agents should be considered in sperm freezing when faced with different quality spermatozoa.
